# Sex-inducing effect of a hydrophilic fraction on reproductive switching in the planarian *Dugesia ryukyuensis *(Seriata, Tricladida)

**DOI:** 10.1186/1742-9994-8-23

**Published:** 2011-10-17

**Authors:** Kazuya Kobayashi, Motonori Hoshi

**Affiliations:** 1 Center for Integrated Medical Research, School of Medicine, Keio University, 35 Shinjuku-ku, Tokyo, 160-8582, Japan; 2 The Open University of Japan, 2-11 Wakaba, Mihama-ku, Chiba, 261-8586, Japan

**Keywords:** Reproductive strategy, asexual reproduction, sexual reproduction, sex-inducing substance, *Dugesia ryukyuensis*, planarians

## Abstract

**Background:**

The mechanisms underlying the switching from an asexual to a sexual mode of reproduction, and *vice versa*, remain unknown in metazoans. In planarians, asexual worms acquire cryptic sexuality when fed with sexual worms, indicating that sexual worms contain a sex-inducing substance. Although such a chemical compound will provide clues about the mechanisms underlying the switching, information on the sex-inducing substance is poor. In order to identify this substance, we have established an assay system for sexual induction in asexual worms of *Dugesia ryukyuensis *by feeding them with sexual worms. Here, we carried out an isolation study on the sex-inducing substance using this assay system.

**Results:**

After centrifugation of sexual worms homogenised in saline solution, we found that not only did the precipitate have a sex-inducing effect on the asexual worms, which has been shown previously, but the cytosolic fraction did as well. We confirmed that the sex-inducing activity in the cytosolic fraction was recovered in a hydrophilic fraction separated on an octadecylsilane (ODS) column. We showed that the sex-inducing substance in the hydrophilic fraction is papain-resistant and a putative low-molecular-weight compound of less than 500. We also suggest the presence of an enhancer of sexual induction with a molecular weight (MW) of more than 5 K in the hydrophilic fraction.

**Conclusion:**

Our experiments showed the existence of a sex-inducing substance and an enhancer of sex-induction in a hydrophilic fraction, and a putative hydrophobic sex-inducing substance in the precipitate. Sexual induction in the asexual worms might be triggered by additive or synergistic effects of these chemical compounds.

## Background

Many metazoans can reproduce sexually as well as asexually. When environmental factors are favourable, asexual reproduction is employed to exploit suitable conditions for survival. In general, they switch from an asexual to a sexual mode of reproduction, when individual survival is greatly hampered. Variations found in the offspring resulting from sexual reproduction allow some individuals to be better suited for survival and provide a mechanism for selective adaptation. Therefore, the reproductive strategy of switching between asexual and sexual reproduction may contribute to fitness. However, the mechanisms underlying the switch between the 2 modes remain unknown.

Some freshwater planarians (Platyhelminthes, Turbellaria, Seriata, Tricladida) reproduce asexually as well as sexually [1-6]. The sexual worms are hermaphrodites, while the asexual worms reproduce by fission without forming any sexual organs. Certain worms develop sexual organs during the colder season, while they reproduce asexually with much degenerated gonads during the warmer season of the year. Asexual worms can switch to the sexual mode if they are fed sexual worms; this means that the sexual worms contain a sex-inducing substance that is not species-specific [7,8]. The putative sex-inducing substance is likely to provide clues about the mechanisms underlying the switch from an asexual to a sexual mode of reproduction. However, very little information is available on the sex-inducing substance. Grasso *et al*. [9] tried to purify a sex-inducing substance contained in *Polycelis nigra *by using asexual worms of *Dugesia gonocephala *as test worms. They homogenised *P. nigra *in saline solution, and then 3 fractions of the precipitate and 1 fraction of the supernatant were obtained after a 3-step centrifugation. Sex-inducing activity was detected only in the first and second precipitates but not in the supernatant (the cytosolic fraction). This suggested that the sex-inducing substance contained in *P. nigra *might be a hydrophobic compound.

In order to isolate and identify this sex-inducing substance, we have established a bioassay system for sexual induction. We planned to use asexual worms exclusively as the test worms for isolating and identifying the sex-inducing substance. Other important issues regarding the animals were the supply (abundance, constancy, ease, and readiness), easy culture in laboratory, resistance to operations such as ablation, and adequate body size. Considering all these points, we chose the OH strain of *Dugesia ryukyuensis *as the test worms because spontaneous sexual induction has never been observed in this strain under laboratory conditions. No functional sexual organs were externally recognised in the test worms before or after they consumed conspecific asexual worms and chicken liver — their daily food. However, when they were fed the minced worms of *B. brunnea*, an oviparous species, they developed hermaphroditic sexual organs. After an intensive and extensive survey of the appropriate conditions of the bioassay, such as the population density, feeding procedure, and temperature, we established a simple, reliable, and a relatively quick assay system [10-14]. We divided the process of sexual induction into 5 distinct stages by morphological changes [[10,12], Additional file 1]. In stage 1, the ovaries became sufficiently large to be externally apparent behind the head, yet no oocytes or other sexual organs were detectable. In stage 2, oocytes appeared in the ovaries, but other sexual organs remained undetectable. In stage 3, the primordial testes emerged and a copulatory apparatus became visible as a white speck in the post-pharyngeal region. In stage 4, yolk gland primordia developed, and spermatocytes appeared in the testes. In stage 5, matured yolk glands formed, and many matured spermatozoa were detectable in the testes. We also found that sexual induction has a point-of-no-return between stages 2 and 3. The test worms at stages 1 and 2 return to being asexual if feeding on *B. brunnea *is stopped. On the contrary, the test worms at stages 3 onward keep developing sexual organs, even though feeding on *B. brunnea *is stopped. Briefly, by external observation, we can recognise the test worms with only a pair of ovaries and those with a copulatory apparatus as the state before and after the point-of-no-return, respectively. Furthermore, *Dryg *is expressed from the primordial to the mature yolk glands at stages 3 onward, which means that it can be used as a marker gene for the degree of sexual induction after the point-of-no-return [15].

In the present study, we used sexual worms of *D. ryukyuensis *as a source of the sex-inducing substance. Sex-inducing activity was examined by external observation and *Dryg *expression. After centrifugation, we found that both the cytosolic fraction and the precipitate displayed sex-inducing activity in the asexual worms. Furthermore, we recovered and analysed the sex-inducing activity in a more hydrophilic fraction (fraction M0 + M10) by using a commercial octadecylsilane (ODS) column. We have not been able to establish if more than 1 substance is responsible for sexual induction, but until this is resolved, we will refer to the substance in singular.

## Results

### Sex-inducing activity in the precipitates from samples extracted with phosphate-buffered saline

**Figure 1:**
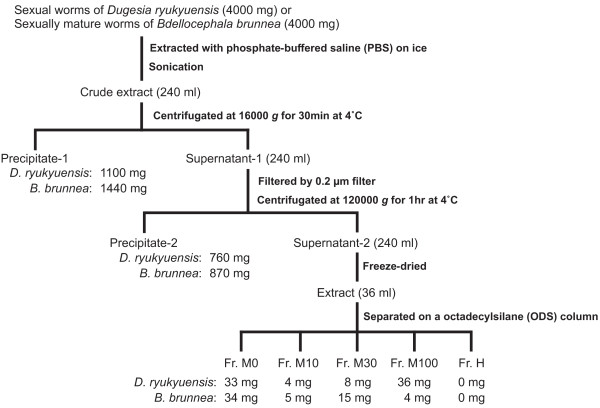
**Procedure for the fractionation of *Dugesia ryukyuensis *and *Bdellocephala brunnea***. See the text for experimental details. Fr., fraction.

Because the test worms avoided consuming minced sexual worms of the OH strain, we could not estimate the specific activity (purification) of the sex-inducing substance in the sexual worms of *D. ryukyuensis *by using the established purification procedure (Figure 1). To estimate the specific activity, we instead used *B. brunnea *as a source of the sex-inducing substance. About 400 mg of *B. brunnea *was sufficient to induce the sexual mode of reproduction in 25 test worms, when the test worms were fed with minced worms of *B. brunnea *(Table 1). Following Grasso *et al*. [9], we homogenised 4 g of worms in phosphate-buffered saline (PBS) and then obtained 2 fractions of the precipitates after a 2-step centrifugation (Figure 1). Most test worms developed a pair of ovaries, but no copulatory apparatus, when they were fed about 400 mg of these precipitates (Table 1). This suggested that even after ultracentrifugation, the sex-inducing substance was still contained in the cytosolic fraction.

**Table 1:** Sex-inducing activity of the precipitates from samples extracted with phosphate-buffered saline (PBS)

Test food		Number of test worms that developed a pair of ovaries (%)	Number of test worms that developed a copulatory apparatus (%)
*Dugesia ryukyuensis*	ppt-1	44/49 (89.8 ± 2.3)	1/49 (2.0 ± 2.0)
	ppt-2	21/50 (42.0 ± 6.0)	0/50 (0.0 ± 0.0)

*Bdellocephala brunnea*	ppt-1	36/50 (72.0 ± 12.0)	0/50 (0.0 ± 0.0)
	ppt-2	4/49 (8.1 ± 3.9)	0/49 (0.0 ± 0.0)
	Minced worms	49/49 (100.0 ± 0.0)	47/49 (96.0 ± 4.0)

The precipitates were obtained as described in Figure 1. Twenty-five test worms were fed approximately 400 mg of each sample for 4 weeks. Means and standard errors of duplicated experiments are shown. ppt, precipitate.

### Sex-inducing activity of extracts fractioned on the ODS column

**Figure 2:**
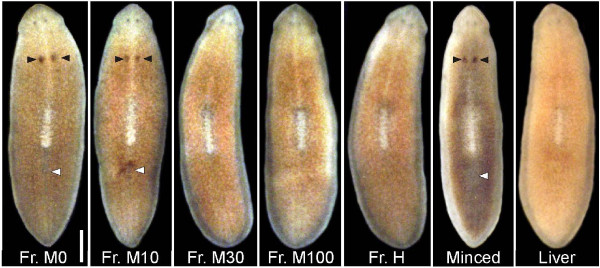
**External sexual appearances of test worms fed *Dugesia ryukyuensis *extracts as shown in Table 2**. Ventral views of test worms at the fourth week of feeding are shown. Test worms were fed fractions M0 (Fr. M0), M10 (Fr. M10), M30 (Fr. M30), M100 (Fr. M100), H (Fr. H), minced *Bdellocephala brunnea *(Minced), and chicken liver (Liver). Arrowheads represent an ovary pair (black) and a copulatory apparatus (white). The images are arranged with the anterior to the top. Scale bars represent 2 mm.

**Figure 3:**
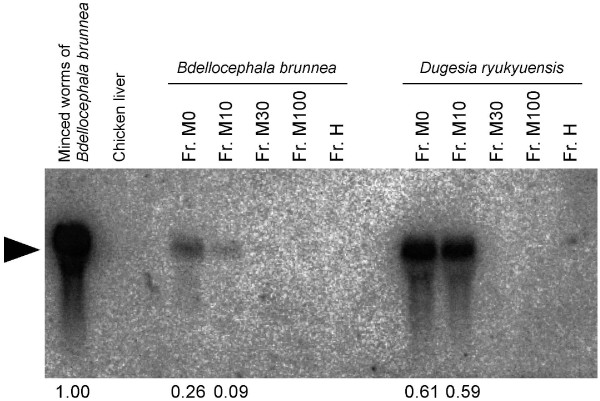
**Quantification of the sex-inducing activity by northern blot analysis of *Dryg***. Ten test worms each were used for total RNA extraction after the feeding assay shown in Table 2. The bands (black arrowhead) were quantified using the programme ImageJ, and the relative values are shown at the bottom of the figure. Fr., fraction.

The cytosolic fraction was desalted and separated on a commercial octadecylsilane (ODS) column (Figure 1). We could not confirm whether the flow-through fraction contained a sex-inducing substance, because the test worms could not eat it due to a high salt concentration. Strong sex-inducing activity was recovered in the fractions eluted with water and 10% methanol (M0 and M10, respectively; Figure 2 and Table 2). This indicates that sexual planarians have a hydrophilic sex-inducing substance. The weight of the M0 and M10 fractions was almost the same between the sexual worms of *D. ryukyuensis *and *B. brunnea *(Figure 1). The sex-inducing activity of the M0 and M10 fractions was expressed as relative values of *Dryg *expression based on minced worms of *B. brunnea *(400 mg; Figure 3). In the sexual worms, the sex-inducing activity per weight of these fractions was 2.3- and 6.6-fold higher than that of *B. brunnea*, respectively. This means that the quality, quantity, or both of the sex-inducing substance in *D. ryukyuensis *differs from the substance in *B. brunnea*. The M0 and M10 fractions were derived from 4 g of *B. brunnea*. Because 25 test worms cannot finish eating 4 g on this assay schedule, the sex-inducing activity of 4 g of *B. brunnea *was extrapolated from that based on consumption of 400 mg. The sex-inducing activity was increased approximately 3- and 7-fold in the M0 and M10 fractions, respectively, using the purification procedure established (Table 3).

**Table 2:** Sex-inducing activity of the extracts separated on a commercial octadecylsilane (ODS) column

Test food		Number of test worms that developed a pair of ovaries (%)	Number of test worms that developed a copulatory apparatus (%)
*Dugesia ryukyuensis*	Fr. M0	50/50 (100.0 ± 0.0)	50/50 (100.0 ± 0.0)
	Fr. M10	47/49 (96.0 ± 4.0)	44/49 (89.5 ± 6.5)
	Fr. M30	0/48 (0.0 ± 0.0)	0/48 (0.0 ± 0.0)
	Fr. M100	0/48 (0.0 ± 0.0)	0/48 (0.0 ± 0.0)
	Fr. H	0/50 (0.0 ± 0.0)	0/50 (0.0 ± 0.0)

*Bdelloceplala brunnea*	Fr. M0	50/50 (100.0 ± 0.0)	44/50 (88.0 ± 8.0)
	Fr. M10	45/49 (91.8 ± 4.3)	39/49 (79.6 ± 4.5)
	Fr. M30	0/50 (0.0 ± 0.0)	0/50 (0.0 ± 0.0)
	Fr. M100	0/48 (0.0 ± 0.0)	0/48 (0.0 ± 0.0)
	Fr. H	0/50 (0.0 ± 0.0)	0/50 (0.0 ± 0.0)
	Minced worms	49/49 (100.0 ± 0.0)	47/49 (96.0 ± 4.0)

Chicken liver		0/50 (0.0 ± 0.0)	0/50 (0.0 ± 0.0)

The extracts were obtained as described in Figure 1. See Materials and methods for test food. Twenty-five test worms were also fed approximately 400 mg of minced worms and chicken liver, respectively, for 4 weeks. Means and standard errors of duplicated experiments are shown. Fr., fraction.

**Table 3:** Purification of a sex-inducing substance in the fractions M0 and M10

Samples	Total weight (mg)	Sex-inducing activity	Specific activity	Purification (fold)
Minced worms	4000	10	0.0025	1×
Fr. M0	34	0.258	0.0076	3×
Fr. M10	5	0.088	0.0176	7×

Sex-inducing activity was represented as relative values of the expression of *Dryg *based on minced worms of *Bdellocephala brunnea *(400 mg) in Figure 3. Fr., fraction.

### Analysis of a sex-inducing effect of the M0 + M10 fraction

To determine the effective concentration of the M0 + M10 fraction for sexual induction, we carried out a dilution experiment (Table 4). Dilutions of 5-fold or higher cannot induce a sexual mode of reproduction in the test worms, but even a 25-fold dilution could cause development of a pair of ovaries in the test worms. Based on this, we set the minimum feeding conditions for the subsequent experiments at 4 g of sexual worms for 25 test animals for 4 weeks.

**Table 4:** Dilution assay of sex-inducing activity in the M0 + M10 fraction of *Dugesia ryukyuensis*

Dilution series	Number of test worms that developed a pair of ovaries (%)	Number of test worms that developed a copulatory apparatus (%)
1	45/50 (90.0 ± 6.0)	38/50 (76.0 ± 16.0)
1/2	40/50 (80.0 ± 4.0)	14/50 (28.0 ± 4.0)
1/5	35/49 (71.5 ± 3.5)	0/49 (0.0 ± 0.0)
1/10	33/50 (62.0 ± 2.0)	0/50 (0.0 ± 0.0)
1/25	3/50 (6.0 ± 6.0)	0/50 (0.0 ± 0.0)
1/125	0/48 (0.0 ± 0.0)	0/48 (0.0 ± 0.0)
1/625	0/50 (0.0 ± 0.0)	0/50 (0.0 ± 0.0)

To prepare test food, each dilution sample was mixed with 150 μl of chicken liver homogenate and then freeze-dried. Twenty-five test worms were fed for 4 weeks. Means and standard errors of duplicated experiments are shown.

**Figure 4:**
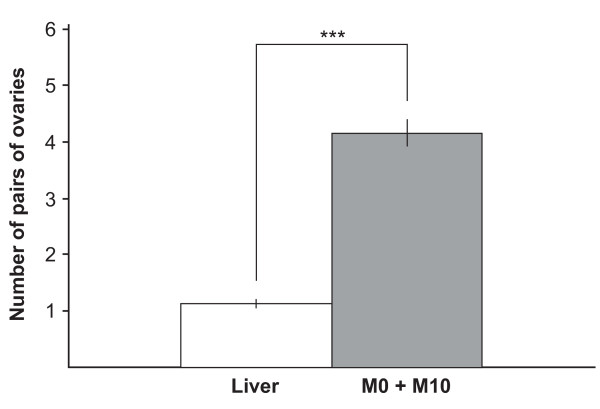
**Induction of supernumerary ovary pairs by feeding with the M0 + M10 fraction**. Test worms with acquired sexuality induced significantly more supernumerary ovary pairs by feeding with the M0 + M10 fraction than that with chicken liver for 2 weeks (Welch's *t*-test: n_M0 + M10 _= 25, n_Liver _= 25; P = 1.59E-12). Error bars represent the standard error. The M0 + M10 fraction of *Dugesia ryukyuensis *was obtained as described in Figure 6. To prepare test food, the M0 + M10 fraction was mixed with 150 μl of chicken liver homogenate and then freeze-dried.

**Figure 5:**
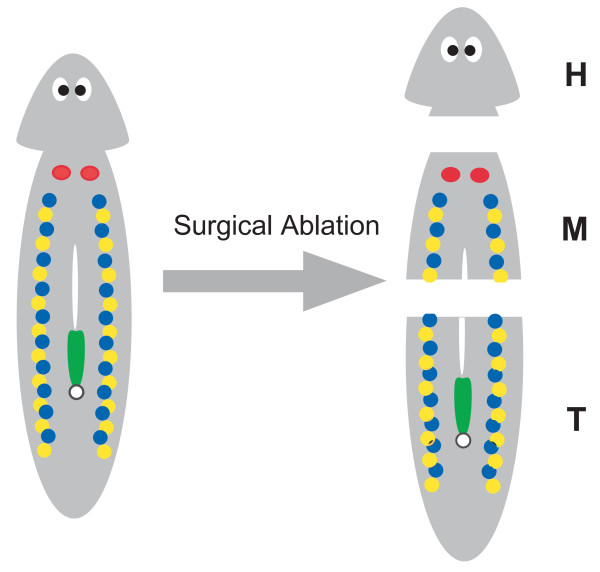
**Scheme of the surgical ablation of sexual worms**. By surgical ablation, 3 different fragments were obtained with respect to the topological position of the sexual organs: the head (H) fragment had no sexual organs; the middle (M) fragment had a pair of ovaries, testes, and yolk glands; the tail (T) fragment had testes, yolk glands, and a copulatory apparatus. Coloured regions correspond with the sexual organs: red, ovary; blue, testis; yellow, yolk gland; green, a copulatory apparatus with a genital pore. The white region in the body is the pharynx. Figure 2 in Kobayashi et al [13] was modified.

Sakurai [8] reported that the asexual worms of *D. japonica *became sexual and developed several supernumerary ovary pairs when fed with *B. brunnea*. We have showed that in the test worms with acquired sexuality, many supernumerary ovary pairs were apparently induced by feeding them with *B. brunnea *(Additional file 2). Supernumerary ovaries were also induced in the test worms with acquired sexuality by feeding them the M0 + M10 fraction of conspecific sexual worms (Figure 4). Sakurai [8] also reported that the feeding of the head portion of *B. brunnea *did not cause complete sexual induction of asexual worms, whereas that of the portions other than the head did cause complete sexual induction. Following Sakurai [8], we fed the test worms the M0 + M10 fractions derived from 3 fragments of conspecific sexual worms (Figure 5). The M0 + M10 fractions from the middle (M) and tail (T) fragments were effective for ovarian induction, but those from the head (H) fragments were ineffective (Table 5). Approximately 70% of the test worms acquired full sexuality after consuming the M0 + M10 fractions from the M and T fragments, whereas no test worms acquired sexuality by feeding on the M0 + M10 fraction from the H fragments (Table 5).

**Table 5:** Sex-inducing activity in 3 fragments obtained according to the topological position of the sexual organs

Test food	Number of test worms that developed a pair of ovaries (%)	Number of test worms that differentiated a copulatory apparatus (%)
Head fragments	0/25 (0.0)	0/25 (0.0)
Middle fragments	24/25 (96.0)	18/25 (72.0)
Tail fragments	24/25 (96.0)	15/25 (60.0)

The different fragments are described in Figure 5.

**Figure 6:**
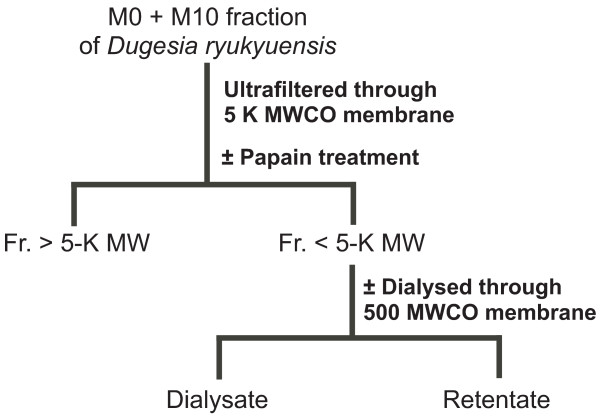
**Procedure for fractionation of the M0 + M10 fraction from *Dugesia ryukyuensis***. See the text for experimental details. Fr., fraction. MW, molecular weight. MWCO, molecular weight cut-off.

**Figure 7:**
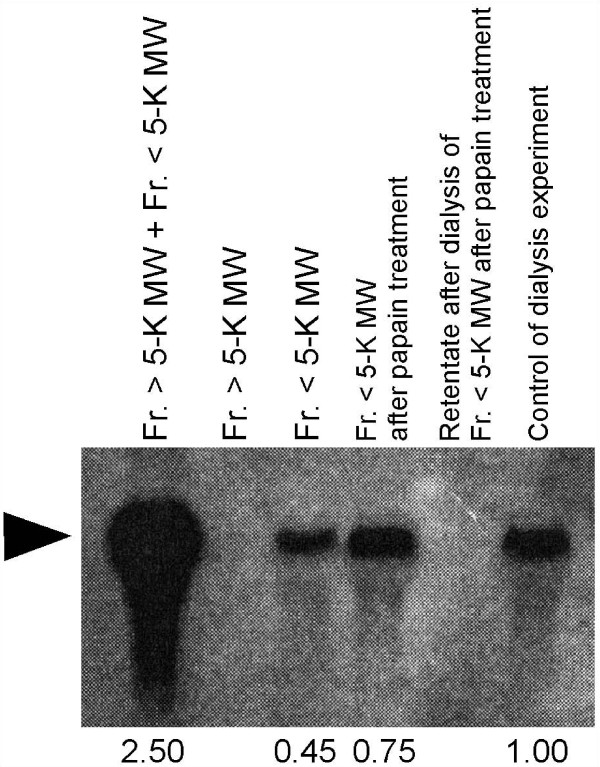
**Comparative analysis of the sex-inducing activity in the M0 + M10 fraction by expression of *Dryg***. Ten test worms each were used for total RNA extraction after the feeding assay in Table 6. The bands (black arrowhead) were quantified using the programme ImageJ, and relative values are shown at the bottom of the figure. Fr., fraction. MW, molecular weight.

When the homogenate of the sexual worms was boiled at 100°C for 15 min, the sex-inducing activity in the M0 + M10 fraction decreased sharply, and the test worms only developed a pair of ovaries (data not shown). To confirm whether the sex-inducing substance contained in the M0 + M10 fraction was a protein, we used a cysteine protease, papain, and ultrafiltration. The procedure used to separate the M0 + M10 fraction is shown in Figure 6. First, we separated the M0 + M10 fraction by ultrafiltration through 5-K molecular weight cut-off (MWCO) membranes, which remove papain (molecular weight [MW]: 22 kD). Sex-inducing activity was only recovered in the fraction of less than 5 K MW (< 5-K MW fraction) (Table 6). Interestingly, evaluation of sex-inducing activity on the basis of *Dryg *expression revealed that the < 5-K MW fraction showed a 5.6-fold increase in sex-inducing activity by the addition of the > 5-K MW fraction, even though the > 5-K MW fraction alone did not have any sex-inducing activity (Figure 7). This suggests the existence of an enhancer for sexual induction in the > 5-K MW fraction. Next, we performed protein digestion with papain for the M0 + M10 fraction. The test worms not only developed a pair of ovaries but also a copulatory apparatus when fed the protein-digested < 5-K MW fraction (Table 6), and the sex-inducing activity increased 1.7-fold (Figure 7). Finally, the < 5-K MW fraction after papain treatment was dialysed using approximately 30 L water for 48 h. The otherwise < 5-K MW fraction after papain treatment was placed under the same conditions, except for dialysis, as a control to consider the possibility that the sex-inducing substance is non-specifically degraded (Figure 7 and Table 6). The test worms did not attain complete sexuality after being fed the retentate after dialysis of 500 MWCO, whereas they became sexual when fed the control food (Figure 7 and Table 6). This suggested that the hydrophilic sex-inducing substance is a low-MW compound (MW: < 500). The dialysate should contain the sex-inducing substance. However, we did not examine the dialysate because concentrating the large amount of dialysate (approximately 30 L) was not realistic in terms of an isolation study (effective recovery of the sex-inducing substance).

**Table 6:** Molecular weight estimation and papain susceptibility testing for a sex-inducing substance

Test food	Number of test worms that developed a pair of ovaries (%)	Number of test worms that developed a copulatory apparatus (%)
Fr. > 5-K MW + Fr. < 5-K MW	50/50 (100.0 ± 0.0)	32/50 (64.0 ± 4.0)
Fr. > 5-K MW	0/50 (0.0 ± 0.0)	0/50 (0.0 ± 0.0)
Fr. < 5-K MW	49/50 (98.0 ± 2.0)	28/50 (56.0 ± 4.0)
Fr. < 5-K MW after papain treatment	47/50 (94.0 ± 6.0)	32/50 (64.0 ± 4.0)
Retentate after dialysis of papain-treated Fr. < 5-K MW	4/50 (8.0 ± 8.0)	0/50 (0.0 ± 0.0)
Control of dialysis experiment	50/50 (100.0 ± 0.0)	27/50 (54.0 ± 2.0)

The M0 + M10 fraction of *Dugesia ryukyuensis *was obtained as described in Figure 6. See Materials and methods for test food. Control of dialysis experiment: the < 5-K molecular weight (MW) fraction after papain treatment was placed under the same conditions except for dialysis. Means and standard errors of duplicated experiments are shown. Fr., fraction.

## Discussion

The existence of a sex-inducing substance was demonstrated by Grasso and Benazzi [7]. Grasso *et al*. [9] reported that sex-inducing activity was recovered in the precipitate, but not in the cytosolic fraction, obtained after centrifugation of the homogenate of sexual worms in saline solution. Since then, there has been no substantial improvement in the isolation of a sex-inducing substance from sexual worms. We tried to purify the sex-inducing substance contained in *D. ryukyuensis *by using conspecific sexual worms. Following Grasso *et al*. [9], we homogenised 4 g of sexual worms (about 320 worms) in phosphate-buffered saline (PBS) and then obtained 2 fractions of the precipitate after 2-step centrifugation (Figure 1). The test worms did not attain complete sexuality when fed precipitates 1 and 2, although they did develop a pair of ovaries (Table 1). Similarly, feeding the test worms with ~400 mg of *B. brunnea *precipitates also resulted in the development of a pair of ovaries only. Because ~400 mg of minced worms of *B. brunnea *was sufficient to induce a sexual mode of reproduction in 25 test worms, it was expected that the cytosolic fraction contained the sex-inducing substance. In *P. nigra*, sex-inducing activity was recovered only in the precipitate [9], and the responsible substance might be a hydrophobic compound. In order to confirm whether the sex-inducing substance in the cytosolic fraction of *D. ryukyuensis *is hydrophobic or hydrophilic, we separated the cytosolic fraction by using a commercial octadecylsilane (ODS) column. Strong sex-inducing activity was recovered in the fractions eluted with water and 10% methanol (fractions M0 and M10, respectively) (Figures 2 and 3, Table 2). This means that the sex-inducing substance contained in *D. ryukyuensis *is a hydrophilic as well as a hydrophobic compound.

Although sex-inducing activity in the M0 + M10 fraction was extremely decreased when the homogenate of the sexual worms was boiled at 100°C for 15 min, the test worms did develop a pair of ovaries (data not shown). In addition, although a 2-fold diluted M0 + M10 fraction was sufficient to induce a sexual mode of reproduction in the test worms, even a 25-fold dilution could cause development of a pair of ovaries in the test worms (Table 4). This suggests that the M0 + M10 fraction contains an ovary-inducing substance that can operate partially independently of other substances involved in acquiring complete sexuality. We found a seasonal change in the quality, quantity, or both, of the sex-inducing substance contained in *B. brunnea *[14]. When *B. brunnea *were immature in summer, the sex-inducing activity of the test worms was extremely low. The feeding with *B. brunnea *frozen in summer induced merely a pair of ovaries in spite of the long-term feeding. On the contrary, when they were sexually mature in winter, the sex-inducing activity was higher. This implied the existence of the ovary-inducing substance. Conversely, the test worms with acquired sexuality developed many supernumerary ovary pairs after consuming *B. brunnea *and the M0 + M10 fraction of conspecific sexual worms (Figure 4, Additional file 2). The supernumerary ovaries would be induced by an overdose of the ovary-inducing substance.

Previously, we concluded that an action of the sex-inducing substance induces the production of the sex-inducing substance in otherwise asexual worms. Once the worms acquired sexuality, they gained the ability to produce the sex-inducing substance [13]. It was suggested that the head portion of *B. brunnea *lacked some of the sex-inducing substances necessary for the complete sexual induction of asexual worms, whereas the portions other than the head contained sufficient amounts of the sex-inducing substance [8]. Following Sakurai [8], the test worms with acquired sexuality were cut into 3 pieces, and the 3 fragments were allowed to regenerate. The head (H) fragments regenerated to become asexual, whereas the middle (M) and tail (T) fragments regenerated to become sexual [[13], Additional file 3]. In this study, we fed the test worms the M0 + M10 fractions derived from the 3 fragments of conspecific sexual worms (Table 5). The activity of the sex-inducing substance in the M0 + M10 fraction was recognised in the M and T fragments but not in the H fragments. These results strongly suggested that the sex-inducing substance in the M0 + M10 fraction is involved in the maintenance of acquired sexuality.

The test worms became fully sexual by being fed the < 5-K MW fraction after protein digestion with papain of the M0 + M10 fraction (Table 6). The putative MW of this sex-inducing substance was estimated at less than 500 by the dialysis experiment (Figure 7 and Table 6). Thus, the sex-inducing substance contained in the M0 + M10 fraction is probably not a protein, although we cannot completely deny that the sex-inducing substance is a protein or a peptide lacking papain-recognition sites. We have also found that the > 5-K MW fraction clearly enhanced the sex-inducing activity of the < 5-K MW fraction, although the > 5-K MW fraction did not have any inherent sex-inducing activity (Figure 7 and Table 6). This indicates that the > 5-K MW fraction probably contains enhancers for the sex-inducing activity of the < 5-K MW fraction. Interestingly, papain treatment of the M0 + M10 fraction seemed to enhance the sex-inducing activity of the < 5-K MW fraction (Figure 7).

The sex-inducing substance might contribute to an understanding of the mechanisms underlying the switch from an asexual to a sexual mode of reproduction in planarians. In the multicellular green flagellate *Volvox carteri*, heat shock elicits the production of a sexual inducer, a 20-kD glycoprotein [16-18]. This protein causes their asexual reproductive cells, called gonidia, to undergo a modified pattern of embryonic development, resulting in the production of gametes, depending on the genetic sex of the individual [19]. However, such compounds have not been reported in metazoans. We suggest that one of the planarian sex-inducing substances could be a hydrophilic compound with a low MW, i.e. less than 500. Sexual planarians seem to contain steroids such as testosterone and estradiol [20,21]. Although these steroids are low-MW compounds, they are rather hydrophobic. Indeed, the test worms did not exhibit any sign of sexual induction when fed these steroids (from 1 ng (approximately 1.43 pg·day^-1^·worm^-1^) to 1 μg (approximately 1.43 ng·day^-1^·worm^-1^)) under the bioassay conditions in this study. Thus, these steroids are not the sex-inducing substance. Recently, it has been reported that a neuropeptide Y (NPY) superfamily member plays an important role in the normal development and maintenance of the planarian reproductive system in *Schmidtea mediterranea *[22]. Particularly, *npy-8 *is expressed in sexual but not asexual worms. RNAi treatment for *npy-8 *facilitated regression of the reproductive organs. The predicted peptide size of NPYs is approximately 30 amino acid residues (approximately 3 K MW) [23,24]. In terms of MW, NPYs are inconsistent with our putative sex-inducing substance. If the sex-inducing substance is a peptide, it might be another neuropeptide of about 2-4 amino acid residues.

Sex-inducing activity in the M0 and M10 fractions was increased only about 3- and 7-fold, respectively (Table 3). Probably, the precipitates contain a large amount of the hydrophobic sex-inducing substance (Figure 1 and Table 1). Sexual induction might be triggered by additive or synergistic effects of the hydrophobic and the hydrophilic sex-inducing substance, including the enhancer. Thus, in terms of purification of the hydrophilic sex-inducing substance, the procedure established is useful. In the near future, we expect that a sex-inducing substance will be isolated by HPLC purification in the bioassay system outlined in the present study and identified by NMR analysis.

## Conclusions

We greatly improved the purification procedure of a planarian sex-inducing substance with our bioassay system. We showed that sex-inducing activity was recovered in the cytosolic fraction as well as in the precipitate. We clearly showed that the sex-inducing substance in the cytosolic fraction is hydrophilic, papain-resistant, and a putative low-MW compound of less than 500. We also suggested the presence of an enhancer of sexual induction in the hydrophilic fraction. We conclude that sexual induction might be triggered by additive or synergistic effects of the hydrophobic sex-inducing substance in the precipitate and the sex-inducing substance and its enhancer in the hydrophilic fraction.

## Materials and methods

### Animals

An exclusively asexual strain, the OH strain, of the planarian *D. ryukyuensis *[25,26] was maintained at 20°C in dechlorinated tap water and fed chicken liver once a week. The worms of this strain were used as test animals for sexual induction. Wild populations of *B. brunnea*, an oviparous planarian, were collected in the vicinity of Yamagata City, Japan, and then frozen in liquid nitrogen and stored at -80°C. The sexual worms of *D. ryukyuensis *were obtained by feeding the worms of the OH strain with *B. brunnea *[27], as described previously [10]. The sexual worms of *D. ryukyuensis *were cut and allowed to regenerate. They were maintained at 20°C in dechlorinated tap water and fed chicken liver once a week, until maturity. Then, they were stored at -80°C as a source of the sex-inducing substance.

### Preparation of the extracts from sexual worms

Approximately 320 intact worms of *D. ryukyuensis *(4 g in wet weight) and approximately 65 intact worms of *B. brunnea *(4 g in wet weight) were frozen in liquid nitrogen and kept at -80°C until use. Additionally, sexual worms of *D. ryukyuensis *were cut into 3 pieces with a razor. By surgical ablation, 3 different fragments were obtained with respect to the topological position of the sexual organs: the H fragment had no sexual organs; the M fragment had a pair of ovaries, testes, and yolk glands; and the T fragment had testes, yolk glands, and the copulatory apparatus (Figure 5). Pooled frozen fragments (4 g in wet weight; H fragments from approximately 2000 worms, and M and T fragments from approximately 600 worms) were prepared. Pooled frozen samples were homogenised in 240 mL of PBS (34 mM NaCl, 0.68 mM KCl, 2.5 mM Na_2_HPO_4_, and 0.45 mM KH_2_PO_4_; pH 7.4). The homogenate was centrifuged at 16 000 × *g *for 30 min at 4°C. The precipitates were stored at -80°C until the bioassay. The supernatants were filtrated using a 0.2 μm filter (CORNING, Lowell, MA) and then centrifuged at 120 000 × *g *for 1 h at 4°C. The precipitates were stored at -80°C until the bioassay. The supernatants were loaded onto a Sep-Pak^® ^Light tC_18 _Cartridge (Waters, Milford, MA) and eluted with 0%, 10%, 30%, and 100% aqueous methanol and hexane to give the following fractions: M0, M10, M30, M100, and H, respectively (Figure 1). Each fraction was dried, mixed with 150 μl of chicken liver homogenate, freeze-dried, and used as test food for sexual induction. Twenty-five test worms were fed daily on a piece of food over 4 weeks in standard experiments.

### Estimation of sex-inducing activity

To evaluate the degree of sexual induction in the test worms, weekly external observations were carried out under a binocular microscope, specifically paying attention to the development of a pair of ovaries and a copulatory apparatus. Furthermore, we examined the expression for *Dryg *by northern blotting. The total RNA of the test worms was prepared using the guanidinum isothiocyanate/phenol-chloroform method [28]. To analyse the RNA fragments by northern blotting [29], we separated 7.5 μg of total RNA on a 1% agarose gel containing formaldehyde, and transferred them to a positively charged nylon membrane. Antisense P^32^-labelled cDNA probes were prepared with a random prime-labelling system (Amersham Pharmacia Biotech, Schenectady, NY). Hybridisation was carried out at 42°C for 16 h in the hybridisation solution (4× standard sodium citrate [SSC], 50% [v/v] formamide, 0.2% [w/v] sodium dodecyl sulfate [SDS], 5× Denhardt's solution, and 0.12 mg/mL salmon sperm DNA). Post-hybridisation washing was carried out at 50°C for 10 min in 2× SSC-0.1% SDS. Then, the signals were detected with a BAS 5000 Bio-Image Analyzer (Fuji Photo Film, Japan). The bands from the northern blots were quantified using the programme ImageJ 1.44.

### Papain digestion, ultrafiltration experiment, and dialysis experiment for the M0 + M10 fraction

The M0 + M10 fraction was obtained as shown in Figure 1 and separated as shown in Figure 6. It was digested at 37°C for 12 h in 10 μg/mL of papain (SIGMA-ALDRICH, St. Louis, MO) in 1/10 PBS, after which it was ultrafiltrated using a Vivaspin 20-5 K (GE Healthcare, Little Chalfont, UK). The < 5 K molecular weight (MW) fraction was dialysed at 4°C for 48 h using 500 molecular weight cut-off (MWCO) dialysis membranes (Spectra/Por^® ^Biotech CE Membrane; SPECTRUM, Rancho Dominguez, CA). Each sample was freeze-dried, mixed with 120 μl of chicken liver homogenate, freeze-dried again, and used as test food for sexual induction. Twenty-five test worms were fed daily on a piece of food over 3 weeks.

### Statistics

The sample size in the feeding treatments with the M0 + M10 fraction was 25. The induction of supernumerary ovary pairs was evaluated using the Welch's *t*-test.

## Competing interests

The authors declare that they have no competing interests.

## Authors' contributions

The experiment was designed and discussed by KK and MH. Data acquisition, analysis, and drafting of the manuscript were done by KK, with critical revision and major improvements by MH. All authors read and approved the final manuscript.

## Supplementary Material

Additional file 1 **Illustration of 5 distinct stages along with the sexual induction**. The development and topological position of reproductive organs are shown. Coloured regions correspond with the sexual organs: red, ovary; blue, testis; yellow, yolk gland; green, a copulatory apparatus with a genital pore. The white region in the body is the pharynx. Briefly, in stage 1, the ovaries became sufficiently large to be externally apparent behind the head, yet no oocytes or other sexual organs were detectable. In stage 2, oocytes appeared in the ovaries, but other sexual organs remained undetectable. In stage 3, the primordial testes emerged and a copulatory apparatus became visible as a white speck in the post-pharyngeal region. In stage 4, yolk gland primordia developed, and spermatocytes appeared in the testes. In stage 5, matured yolk glands formed, and many matured spermatozoa were detectable in the testes. Figure 1 in Kobayashi and Hoshi [12] was modified. Click here for file

Additional file 2 **Induction of supernumerary ovary pairs by feeding with *Bdellocephala brunnea***. Asexual worms of *Dugesia japonica *became fully sexual and induced several supernumerary ovary pairs along the ventral nerve cord and reaching to the pharyngeal level, when they were fed *Bdellocephala brunnea*, an oviparous species [8]. In general, it is believed that sexual planarians (triclads) have only a pair of ovaries behind the head. Usually, sexual worms of *D. ryukyuensis *also have a pair of ovaries when fed chicken liver daily (a laboratory condition). However, sometimes some worms develop a pair of supernumerary ovaries in the condition. Additionally, we collected a few sexual worms of *D. ryukyuensis *with a few supernumerary ovary pairs in a natural habitat (lat 26°34'01.76″N, long 128°02'14.74″E: Oura river, Okinawa Prefecture, Japan). Some *Dugesia *worms might have the ability to produce supernumerary ovary pairs spontaneously. We showed that in the test worms with acquired sexuality, many supernumerary ovary pairs were apparently induced by feeding them with *B. brunnea*. (A) Test worms with acquired sexuality induced significantly more supernumerary ovary pairs by feeding with *B. brunnea *than that with chicken liver for 2 weeks (Welch's *t*-test: n_Minced *B. brunnea *_= 15, n_Liver _= 15; P = 8.44E-06). Error bars represent the standard error. (B-D) Morphological examination of test worms with supernumerary ovary pairs. Ventral view of a test worm with acquired sexuality fed chicken liver (B) and *B. brunnea *(C). Scale bar: 2 mm. Sagittal section of a test worm with supernumerary ovary pairs (D). Scale bar: 500 μm. Arrowheads represent a main ovary (black) and a supernumerary ovary (red); t, testes; y, yolk glands. The images are arranged with the anterior side at the top. Click here for file

Additional file 3 **Reproductive mode after surgical ablation in the test worms with acquired sexuality**. Fifty-six test worms with acquired sexuality were cut as described previously [[13], Figure 5]. Fragments were transferred separately to a plastic dish (diameter, 3 cm) containing dechlorinated tap water, allowed to regenerate there at 20°C, and fed chicken liver once a week. External observations were performed weekly to determine whether regenerated worms became sexual or asexual. Click here for file
