# Supplementary figures and images for: Sex-inducing effect of a hydrophilic fraction on reproductive switching in the planarian Dugesia ryukyuensis (Seriata, Tricladida)
Source: Front Zool. 2011 Oct 17;8:23. doi: 10.1186/1742-9994-8-23 (PMC3215924; doi:10.1186/1742-9994-8-23)

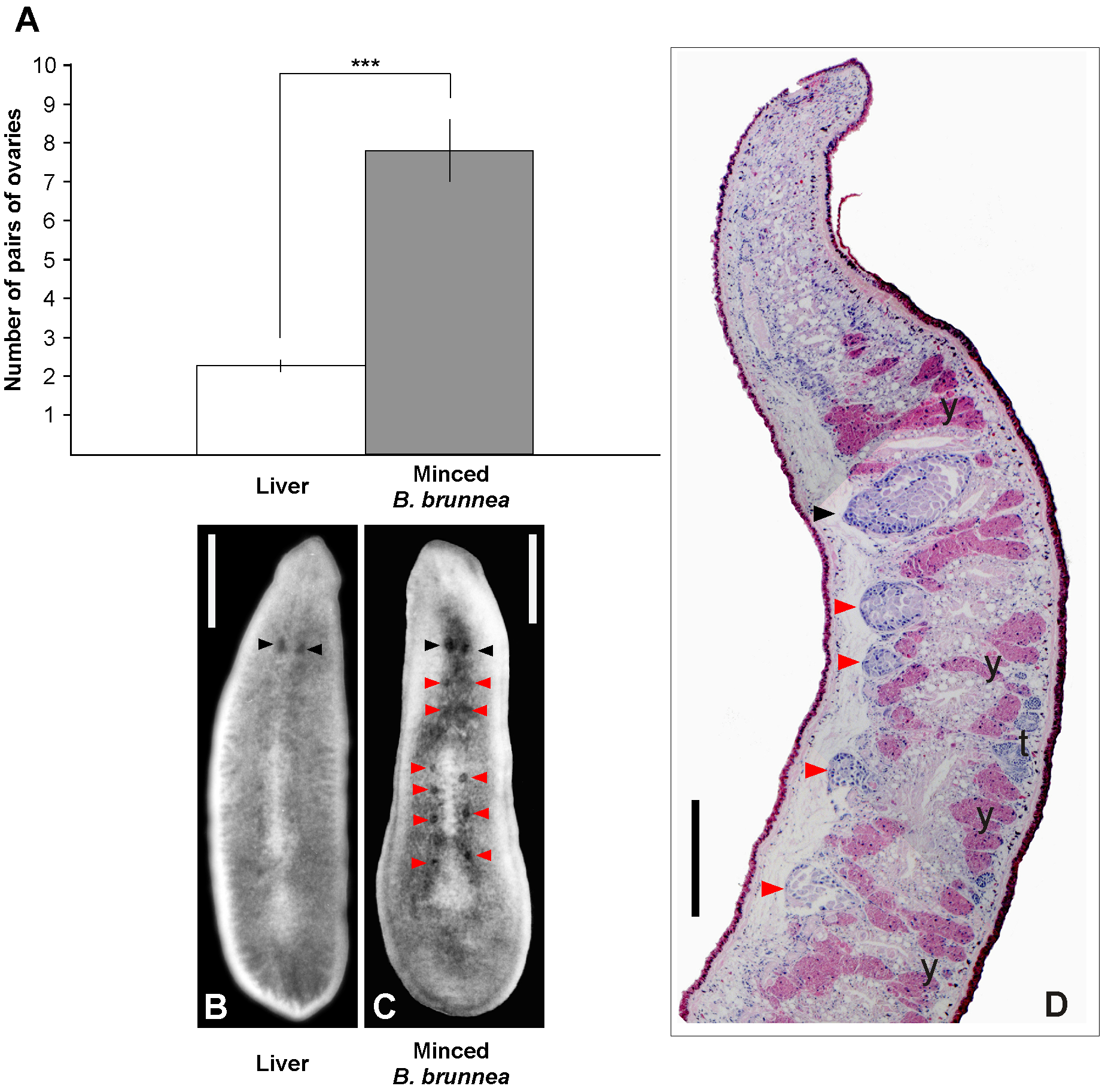

Supplement: Additional file 2 — Induction of supernumerary ovary pairs by feeding with Bdellocephala brunnea. Asexual worms of Dugesia japonica became fully sexual and induced several supernumerary ovary pairs along the ventral nerve cord and reaching to the pharyngeal level, when they were fed Bdellocephala brunnea, an oviparous species [8]. In general, it is believed that sexual planarians (triclads) have only a pair of ovaries behind the head. Usually, sexual worms of D. ryukyuensis also have a pair of ovaries when fed chicken liver daily (a laboratory condition). However, sometimes some worms develop a pair of supernumerary ovaries in the condition. Additionally, we collected a few sexual worms of D. ryukyuensis with a few supernumerary ovary pairs in a natural habitat (lat 26°34'01.76″N, long 128°02'14.74″E: Oura river, Okinawa Prefecture, Japan). Some Dugesia worms might have the ability to produce supernumerary ovary pairs spontaneously. We showed that in the test worms with acquired sexuality, many supernumerary ovary pairs were apparently induced by feeding them with B. brunnea. (A) Test worms with acquired sexuality induced significantly more supernumerary ovary pairs by feeding with B. brunnea than that with chicken liver for 2 weeks (Welch's t-test: nMinced B. brunnea = 15, nLiver = 15; P = 8.44E-06). Error bars represent the standard error. (B-D) Morphological examination of test worms with supernumerary ovary pairs. Ventral view of a test worm with acquired sexuality fed chicken liver (B) and B. brunnea (C). Scale bar: 2 mm. Sagittal section of a test worm with supernumerary ovary pairs (D). Scale bar: 500 μm. Arrowheads represent a main ovary (black) and a supernumerary ovary (red); t, testes; y, yolk glands. The images are arranged with the anterior side at the top. [file 1742-9994-8-23-S2.TIFF]
